# Enhanced increase of omega-3 index in healthy individuals with response to 4-week n-3 fatty acid supplementation from krill oil versus fish oil

**DOI:** 10.1186/1476-511X-12-178

**Published:** 2013-12-05

**Authors:** Vanu R Ramprasath, Inbal Eyal, Sigalit Zchut, Peter JH Jones

**Affiliations:** 1 Richardson Centre for Functional Foods and Nutraceuticals, Winnipeg, Canada; 2 Department of Human Nutritional Sciences, University of Manitoba, Winnipeg, Canada; 3 Enzymotec Ltd. P.O.B 6, Migdal HaEmeq, Israel

**Keywords:** Krill oil, Fish oil, Omega-3 index, Fatty acids, Human

## Abstract

**Background:**

Due to structural differences, bioavailability of krill oil, a phospholipid based oil, could be higher than fish oil, a triglyceride-based oil, conferring properties that render it more effective than fish oil in increasing omega-3 index and thereby, reducing cardiovascular disease (CVD) risk.

**Objective:**

The objective was to assess the effects of krill oil compared with fish oil or a placebo control on plasma and red blood cell (RBC) fatty acid profile in healthy volunteers.

**Participants and methods:**

Twenty four healthy volunteers were recruited for a double blinded, randomized, placebo-controlled, crossover trial. The study consisted of three treatment phases including krill or fish oil each providing 600 mg of n-3 polyunsaturated fatty acids (PUFA) or placebo control, corn oil in capsule form. Each treatment lasted 4 wk and was separated by 8 wk washout phases.

**Results:**

Krill oil consumption increased plasma (p = 0.0043) and RBC (p = 0.0011) n-3 PUFA concentrations, including EPA and DHA, and reduced n-6:n-3 PUFA ratios (plasma: p = 0.0043, RBC: p = 0.0143) compared with fish oil consumption. Sum of EPA and DHA concentrations in RBC, the omega-3 index, was increased following krill oil supplementation compared with fish oil (p = 0.0143) and control (p < 0.0001). Serum triglycerides and HDL cholesterol concentrations did not change with any of the treatments. However, total and LDL cholesterol concentrations were increased following krill (TC: p = 0.0067, LDL: p = 0.0143) and fish oil supplementation (TC: p = 0.0028, LDL: p = 0.0143) compared with control.

**Conclusions:**

Consumption of krill oil was well tolerated with no adverse events. Results indicate that krill oil could be more effective than fish oil in increasing n-3 PUFA, reducing n-6:n-3 PUFA ratio, and improving the omega-3 index.

**Trial registration:**

ClinicalTrials.gov, NCT01323036

## Background

Epidemiological and clinical studies have shown various health benefits with consumption of fish and sea foods [1-6]. These positive health outcomes are attributed to the presence of n-3 polyunsaturated fatty acids (PUFA) in foods, including eicosapentaenoic acid (EPA) and docosahexaenoic acid (DHA). Krill oil, extracted from “*Euphausia Superba*”, a species of the Southern ocean, is a rich source of n-3 PUFA including EPA and DHA. Krill oil was found to be Generally Recognized as Safe (GRAS) by the American Food and Drug Administration (FDA) and obtained a Novel Food status from the European Union. Krill oil is comprised of n-3 PUFA, antioxidant carotenoid astaxanthin, vitamins A and E, phospholipids (PL) as well as various other fatty acids [7,8]. Furthermore, numerous clinical and pre-clinical trials have shown that krill oil is safe and well tolerated, with no indication of adverse effects [9-13]. Consumption of krill oil increases plasma EPA and DHA levels in normal, overweight and obese participants [10].

Pre-clinical studies have shown that absorption of fatty acids attached to PL into target organs such as heart, brain and liver of animals, is better than fatty acids attached to glycerol as triglycerides (TG) [14]. Krill and fish oils differ in their composition where krill oil is comprised of PL and TG and fish oil is comprised of only TG. The primary PL in krill oil is phosphatidylcholine (PC), with 40% of the total fatty acids attached to the PC being EPA and DHA [15].

Results from animal and human studies suggest that krill oil is more effective compared with fish oil in improving CVD risk markers [11,16]. Metabolic effects of krill oil were reported to be similar to those of fish oil but at lower doses of EPA and DHA in healthy volunteers [17]. However, although superior bioavailability of n-3 PUFA in krill oil over fish oil was suggested, none of the studies managed to show improvement in absorption of n-3 fatty acids with krill oil over fish oil. At the most, data from a bioavailability study in humans showed a tendency for higher bioavailability for EPA after krill oil consumption compared with fish oil [18], however, this study tested an acute single dose of n-3 PUFA over 48 h. To the best of our knowledge, no published literature has compared the effects of krill and fish oils after longer term consumption with a larger sample size using a randomized crossover design. Additionally, no randomized controlled trials have been conducted to compare the incorporation of n-3 PUFA to RBC membranes after consumption of krill and fish oil.

The objective of the present trial was therefore to assess the effects of krill oil compared with fish oil and a placebo control (corn oil) on plasma and RBC fatty acid profile in healthy volunteers following 4 wk of supplementation. Specific objectives included assessment and comparison of serum lipid profile in healthy volunteers at baseline and following 4 wk of intervention with krill versus fish oils.

## Results

Thirty four participants (20 males and 14 females) were screened out of which 24 (12 males and 12 females) were eligible and enrolled into the study. All participants completed the study and as such there were no drop outs. Table 1 shows baseline characteristics of the participants involved. Participants were healthy and of age, 28.2 ± 5.4 y with body weight and BMI of 68.3 ± 11.9 (kg) and 23.8 ± 3 (body weight in kg/height in M^2^) respectively. Vital signs including body weight, systolic and diastolic blood pressure and heart rate were within normal ranges. No significant changes were observed for body weight, BMI, waist circumference, systolic and diastolic blood pressure or heart rates after treatment with krill or fish oil compared with control phase (Data not shown).

**Table 1:** Baseline characteristics of participants (n = 24)

**Parameter**	**Mean**	**STD**
**Age (Y)**	28.23	5.35
**Body weight (Kg)**	68.27	11.92
**Height (m)**	1.69	0.07
**BMI (kg/m**^**2**^**)**	23.76	2.96
**Waist (cm)**	78.28	8.25
**Hip (cm)**	92.07	5.18
**Waist/Hip ratio**	0.85	0.08
**Systolic BP (mmHg)**	108.07	10.21
**Diastolic BP (mmHg)**	72.01	7.69
**Heart rate**	69.17	9.28
**TG (mg/dL)**	91.23	36.58
**HDL (mg/dL)**	53.16	13.45
**LDL (mg/dL)**	103.51	22.77
**Total cholesterol (mg/dL)**	175.61	23.39
**WBC count (X10**^**9**^**/L)**	5.93	1.20
**RBC (X10**^**12**^**/L)**	4.70	0.51
**Hematocrit (L/L)**	0.41	0.04
**Hemoglobin (g/L)**	141.33	15.38
**Platelet count (X10**^**9**^**/L)**	219.88	34.53
**Neutrophils (%)**	53.09	7.81
**Lymphocytes (%)**	36.25	7.81
**Monocytes (%)**	8.14	2.16
**Eosinophils (%)**	2.16	1.13
**Basophils (%)**	0.48	0.17
**MCV (fl.)**	88.10	6.74
**MCH (pg)**	30.21	2.69
**MCHC (g/L)**	342.46	6.09

Initial statistical tests that were carried out aimed to test the efficiency of the washout period. No significant differences were seen between baselines of different phases for all parameters measured in plasma and RBC which indicate that the washout period was sufficient to wash out or revert back the levels of n-3 fatty acids in both the plasma and the RBC back to baseline levels.

Table 2 shows the plasma fatty acid profiles of the participants during the study. Increased plasma EPA and DHA levels (p < 0.0001 and p < 0.0002, respectively) were observed with both krill and fish oil treatments compared with control. Krill oil consumption elevated (p = 0.0043) plasma EPA levels compared with fish oil. Plasma concentrations of DPA were found to be elevated after treatment with both krill oil (p = 0.011) and fish oil (p = 0.014) compared with control. Levels of total n-3 fatty acids were also increased (p < 0.0001) after supplementation of krill oil and fish oil compared with control. In addition, krill oil consumption increased the level of total n-3 PUFA compared with fish oil (p = 0.0043). Similar results were obtained for the sum of EPA and DHA where both krill and fish oils increased levels of EPA + DHA in plasma (p < 0.0001) compared with control. Furthermore, krill oil increased the plasma concentrations of EPA + DHA compared with fish oil (p = 0.0011).

**Table 2:** Plasma composition of fatty acids and their changes with different interventions

**Parameter**	**Treatment**	**Baseline**		**End-Point**		**Change**	
		**Mean ± STD**	**P. value**^**1**^	**Mean ± STD**	**P. value**^**1**^	**Mean ± STD**	**P. value**^**1**^
**EPA**	Krill oil	0.80 ± 0.31	0.5134	1.97 ± 0.58^*#^	<0.0001	1.17 ± 0.52^*#^	<0.0001
	Fish oil	0.82 ± 0.36		1.54 ± 0.69^*^		0.71 ± 0.46^*^	
	Corn oil	0.86 ± 0.36		0.79 ± 0.43		-0.06 ± 0.36	
**DPA**	Krill oil	0.61 ± 0.31	0.9087	0.79 ± 0.27^*^	0.0048	0.15 ± 0.23^*^	0.0076
	Fish oil	0.64 ± 0.24		0.68 ± 0.28		0.04 ± 0.18^*^	
	Corn oil	0.64 ± 0.25		0.61 ± 0.16		-0.03 ± 0.19	
**DHA**	Krill oil	2.90 ± 0.58	0.5134	3.86 ± 0.89^*^	<0.0001	0.96 ± 0.69^*^	<0.0001
	Fish oil	2.88 ± 0.59		3.72 ± 0.66^*^		0.84 ± 0.55^*^	
	Corn oil	3.03 ± 0.66		2.80 ± 0.60		-0.24 ± 0.69	
**Total n-3 PUFA**	Krill oil	4.97 ± 0.69	0.8465	7.20 ± 1.35^*#^	<0.0001	2.23 ± 1.13^*#^	<0.0001
	Fish oil	4.96 ± 0.59		6.51 ± 0.97^*^		1.55 ± 0.83^*^	
	Corn oil	5.13 ± 0.95		4.79 ± 0.63		-0.34 ± 0.86	
**EPA + DHA**	Krill oil	3.70 ± 0.63	0.7470	5.83 ± 1.31^*#^	<0.0001	2.13 ± 1.02^*#^	<0.0001
	Fish oil	3.70 ± 0.65		5.26 ± 0.95^*^		1.55 ± 0.73^*^	
	Corn oil	3.89 ± 0.85		3.59 ± 0.59		-0.30 ± 0.76	
**n-6:n-3 ratio**	Krill oil	7.06 ± 1.18	0.5818	4.88 ± 1.37^*#^	<0.0001	-2.17 ± 1.05^*#^	<0.0001
	Fish oil	7.21 ± 0.93		5.32 ± 0.99^*^		-1.89 ± 0.91^*^	
	Corn oil	7.12 ± 1.4		7.57 ± 1.06		0.44 ± 0.95	
**Total SFA**	Krill oil	43.46 ± 1.85	0.3247	43.03 ± 1.63	0.0226	-0.43 ± 1.93	0.0787
	Fish oil	42.92 ± 1.32		43.65 ± 1.06^*^		0.73 ± 1.44	
	Corn oil	42.13 ± 4.74		42.53 ± 2.13		0.40 ± 5.04	
**Total MUFA**	Krill oil	17.23 ± 2.93	0.8825	16.29 ± 2.42^*^	0.0226	-0.94 ± 2.86	0.8465
	Fish oil	16.84 ± 2.47		16.06 ± 1.74^*^		-0.78 ± 2.51	
	Corn oil	17.25 ± 3.06		16.97 ± 2.21		-0.29 ± 3.11	
**Total PUFA**	Krill oil	39.31 ± 2.39	0.2748	40.68 ± 2.06	0.8825	1.36 ± 2.58	0.9592
	Fish oil	40.24 ± 1.98		40.28 ± 1.73		0.05 ± 2.16	
	Corn oil	40.62 ± 3.92		40.51 ± 2.22		-0.11 ± 4.69	
**Total n-6 PUFA**	Krill oil	34.35 ± 2.41	0.6873	33.48 ± 2.26^*^	0.0007	-0.87 ± 2.08	0.0226
	Fish oil	35.28 ± 1.87		33.77 ± 1.75^*^		-1.51 ± 2.07^*^	
	Corn oil	35.49 ± 3.62		35.72 ± 2.14		0.22 ± 4.09	

Values are expressed as percentage of total fatty acids except the n-6:n-3 ratio (n = 24). ^1^Friedman Test was used to test if there is a significant difference between treatment groups regarding the results in baseline, end-point and change. ^*^ and ^#^ indicate significant difference compared with corn and fish oil treatments respectively.

No significant changes in total saturated fatty acids (SFA), PUFA and mono-unsaturated fatty acids (MUFA) concentrations were observed between the three treatments except for an increase in SFA and a decrease in MUFA with fish oil intake (p = 0.0143) compared with control (Additional file 1: Table S1). A decrease in MUFA was also observed after krill oil intake (p = 0.041) compared with control. Total n-6 PUFA concentrations were reduced after both krill (p = 0.0043) and fish (p = 0.0011) oil treatments, compared with control. However, no significant differences were observed in the n-6 PUFA concentrations between krill and fish oil treatments. Both krill and fish oil treatments reduced (p < 0.0001) the ratio between n-6 and n-3 fatty acids compared with control. The ratio of n-6:n-3 fatty acids was found to be reduced (p = 0.0043) by krill oil treatment compared with fish oil treatment.

Table 3 shows the RBC fatty acid profiles of the participants during the study. Red blood cell levels of EPA were elevated (p < 0.0001) following both krill and fish oil treatment compared with control treatment. On the other hand, the RBC EPA level was higher (P = 0.0011) following krill oil treatment compared with fish oil. Krill oil also elevated the RBC levels of DHA (p = 0.0011) compared with control. However, no significant differences existed in the DHA levels after fish oil treatment compared with control. Sum of EPA and DHA in RBC membranes (as percent of total fatty acid content), which is denoted as omega-3 index, increased following krill (p < 0.0001) and fish oil consumption (p = 0.0043) compared with control. Interestingly, krill oil treatment elevated the omega-3 index compared with fish oil treatment (p = 0.0143). The change in omega-3 index after consumption of krill oil was two-fold higher than fish oil (1.04% and 0.47% following krill and fish oil treatments respectively; p = 0.0043). Similarly, krill oil treatment led to an increase in RBC DPA levels compared with fish (p = 0.0412) and control oil (p = 0.0412) supplementation. Treatment with fish oil failed to affect DPA levels in the RBC. Both krill and fish oil treatments reduced (p = 0.0011) total n-6 fatty acid levels compared with control. No differences in the n-6 PUFA concentrations were observed with krill oil compared with fish oil intake. Similar to plasma fatty acid results, no significant changes with RBC total PUFA, MUFA and SFA concentrations were seen between the three treatment phases. As expected, krill and fish oil treatments increased RBC n-3 PUFA levels (p < 0.0001 and p = 0.0143 for krill oil and fish oil respectively) and reduced n-6:n-3 ratio (krill: p < 0.0001, fish: p = 0.0143) compared with control. Krill oil treatment also elevated the total n-3 PUFA concentrations (p = 0.0011) and reduced the n-6:n-3 ratio (p = 0.0143) compared with the fish oil treatment. Total n-6 PUFA was found to be reduced with both krill and fish oil phases (p = 0.0143) compared to control. Furthermore, concentrations of n-6 PUFA were not significantly different between krill and fish oil phases. Additionally, no significant changes in total SFA, PUFA and MUFA concentrations were observed between the three treatments in the RBC (Additional file 1: Table S2).

**Table 3:** RBC composition of fatty acids and their changes with different interventions

**Parameter**	**Treatment**	**Baseline**		**End-Point**		**Change**	
		**Mean ± STD**	**P. value**^**1**^	**Mean ± STD**	**P. value**^**1**^	**Mean ± STD**	**P. value**^**1**^
**EPA**	Krill oil	0.82 ± 0.23	0.8825	1.48 ± 0.38^*#^	<0.0001	0.66 ± 0.29^*#^	<0.0001
	Fish oil	0.80 ± 0.23		1.10 ± 0.25^*^		0.30 ± 0.26^*^	
	Corn oil	0.80 ± 0.21		0.74 ± 0.22		-0.05 ± 0.16	
**DPA**	Krill oil	2.24 ± 0.27	0.7470	2.42 ± 0.21^*#^	0.0421	0.18 ± 0.23	0.0787
	Fish oil	2.29 ± 0.28		2.28 ± 0.24		-0.01 ± 0.29	
	Corn oil	2.25 ± 0.34		2.26 ± 0.27		0.00 ± 0.31	
**DHA**	Krill oil	4.12 ± 0.83	0.2140	4.50 ± 0.75^*^	0.0039	0.38 ± 0.44^*#^	0.0002
	Fish oil	4.16 ± 0.73		4.34 ± 0.83		0.17 ± 0.64	
	Corn oil	4.21 ± 0.89		4.12 ± 0.72		-0.09 ± 0.56	
**Total n-3 PUFA**	Krill oil	7.52 ± 1.03	0.4531	8.74 ± 1.08^*#^	<0.0001	1.22 ± 0.77^*#^	<0.0001
	Fish oil	7.59 ± 0.86		8.04 ± 1.00^*^		0.45 ± 1.00^*^	
	Corn oil	7.59 ± 1.22		7.47 ± 0.95		-0.11 ± 0.90	
**EPA + DHA**	Krill oil	4.94 ± 0.97	0.6873	5.97 ± 1.03^*#^	<0.0001	1.04 ± 0.59^*#^	<0.0001
	Fish oil	4.96 ± 0.79		5.43 ± 0.97^*^		0.47 ± 0.77^*^	
	Corn oil	5.00 ± 1.02		4.86 ± 0.80		-0.14 ± 0.61	
**n-6:n-3 ratio**	Krill oil	4.17 ± 0.68	0.4169	3.45 ± 0.59^*#^	<0.0001	-0.72 ± 0.43^*#^	<0.0001
	Fish oil	4.08 ± 0.47		3.77 ± 0.52^*^		-0.31 ± 0.41^*^	
	Corn oil	4.14 ± 0.84		4.20 ± 0.60		0.06 ± 0.56	
**Total SFA**	Krill oil	43.55 ± 1.98	0.6065	43.12 ± 1.86	0.1969	-0.43 ± 1.86	0.0613
	Fish oil	43.58 ± 1.97		44.22 ± 2.05		0.64 ± 2.26	
	Corn oil	43.62 ± 2.44		43.34 ± 1.51		-0.29 ± 1.92	
**Total MUFA**	Krill oil	18.21 ± 1.17	0.8825	18.55 ± 2.33	0.5134	0.34 ± 2.13	0.3114
	Fish oil	18.17 ± 1.39		17.82 ± 1.31		-0.35 ± 1.03	
	Corn oil	18.34 ± 1.49		18.30 ± 1.32		-0.03 ± 0.93	
**Total PUFA**	Krill oil	38.25 ± 2.33	0.6065	38.33 ± 2.02	0.7470	0.08 ± 1.98	0.5818
	Fish oil	38.25 ± 2.38		37.96 ± 2.45		-0.29 ± 2.59	
	Corn oil	38.05 ± 2.73		38.36 ± 2.03		0.32 ± 2.26	
**Total n-6 PUFA**	Krill oil	30.73 ± 2.01	0.1969	29.59 ± 2.01^*^	0.0107	-1.14 ± 1.66^*^	0.0003
	Fish oil	30.66 ± 1.96		29.92 ± 2.13^*^		-0.74 ± 1.86^*^	
	Corn oil	30.46 ± 2.13		30.89 ± 1.79		0.43 ± 1.65	

Values are expressed as percentage of total fatty acids (n = 24). ^1^Friedman Test was used to test if there is a significant difference between treatment groups regarding the results in baseline, endpoint and change. ^*^ and ^#^ indicate significant difference compared with corn and fish oil treatments respectively.

Serum lipid concentrations of participants during the study are portrayed in Table 4. Serum TG and HDL cholesterol levels did not change across the three treatments. However, total and LDL cholesterol concentrations were found to be higher after krill oil (p = 0.0067; p = 0.0143, for total and LDL cholesterol, respectively) supplementation compared with control. Similarly, fish oil consumption increased total and LDL cholesterol levels (p = 0.0028; p = 0.0143, for total and LDL cholesterol, respectively) compared with control oil supplementation. No differences were observed either in serum total or LDL cholesterol concentrations or in the TC/HDL ratio when comparing krill and fish oil consumption.

**Table 4:** Serum lipid concentrations before and after each treatment

**Parameters**	**Treatment**	**Baseline**	**Endpoint**
		**(mg/dL)**	**(mg/dL)**
TG	Krill oil	101.10 ± 10.53	99.86 ± 8.32
	Fish oil	86.69 ± 6.73	89.20 ± 7.31
	Corn oil	103.41 ± 9.84	103.45 ± 10.53
HDL	Krill oil	55.74 ± 2.91	60.90 ± 2.91
	Fish oil	56.71 ± 2.84	58.81 ± 2.76
	Corn oil	56.22 ± 3.38	58.00 ± 3.07
LDL	Krill oil	93.05 ± 4.66	101.31 ± 6.20^*^
	Fish oil	94.78 ± 5.53	102.43 ± 5.81^*^
	Corn oil	95.03 ± 4.82	93.25 ± 5.29
Total cholesterol	Krill oil	168.85 ± 5.45	181.90 ± 6.75^*^
	Fish oil	169.65 ± 5.60	179.22 ± 5.78^*^
	Corn oil	172.55 ± 5.69	172.24 ± 5.99
	Krill oil	3.21 ± 0.20	3.16 ± 0.22
Total cholesterol/HDL ratio	Fish oil	3.21 ± 0.23	3.24 ± 0.21
	Corn oil	3.29 ± 0.21	3.17 ± 0.21

Values are expressed as Mean ± SEM. n = 24. *denotes significance compared with corn oil treatment.

Overall, no differences were observed between baselines of either the fatty acid tested in each treatment or the order of treatments or placebo received by the participants. In addition, no gender or age effect was found in any of the parameters analyzed. Safety with consumption of krill and fish oil were determined by measuring blood parameters including WBC, RBC, hematocrit, hemoglobin, platelet count, neutrophils, lymphocytes, monocytes, eosinophils, basophils, MCV, MCH and MCHC values. No significant differences were found in any of the above parameters with either krill or fish oil supplementation compared with control (Data not shown).

Medical history and physical examination revealed that none of the participants had any health problems or concerns which are known to affect study outcomes. The study physician recorded and compared the health status of participants at each visit. None of the participants during the study showed major changes in their health status related to the trial.

No adverse or serious adverse events related to the treatments were reported during the period of the study. During the study one participant experienced a skin infection, one had an allergic reaction and another participant had her appendix surgically removed. All these three events occurred during the washout periods and were deemed not related to the study. None of the participants consumed any concomitant medications or supplements that are known to affect study outcomes. All participants reported followed guidelines not to eat more than one serving per mo of fish or sea food. None of the participants exceeded the limit of fish or sea food consumption during intervention or washout periods. Participants consumed the study capsules regularly according to the instructions. Participants were of high compliance with capsule consumption which is evident from the calculated self-reported compliance on capsule consumption. The compliance ratings for capsule consumption during krill and fish oil interventions were 95.79 ± 6.87% and 96.91 ± 5.86%, respectively. During the control oil intervention, the compliance was 96.89 ± 4.27%.

Krill oil treatment failed to produce any gastrointestinal symptoms compared with fish oil or placebo control interventions, except for burping and aftertaste (Table 5). Four participants reported mild and three moderate burping after krill oil consumption, whereas only two participants reported burping (one mild and one moderate) after consuming fish oil or placebo control intervention. Similarly, 7 participants reported having aftertaste (six mild and one moderate) with krill oil consumption. Three participants reported having a mild aftertaste with fish oil consumption, however, none of them felt any aftertaste with control oil consumption. Two participants experienced mild hiccupping during fish oil consumption but no hiccupping was reported with krill oil or placebo control intervention.

**Table 5:** Gastrointestinal responses from participants

**GI symptoms**	** Krill oil**	** Fish oil**	** Corn oil**
	**(No. of participants)**	**(No. of participants)**	**(No. of participants)**
**Hiccup**	-	Mild- 2	-
**Burping**	Mild – 4	Mild – 1	Mild – 1
	Moderate – 3	Moderate – 1	Moderate – 1
**Nausea**	-	-	-
**Vomiting**	-	-	-
**Indigestion**	-	Mild – 1	Mild – 1
**Stomach/abdominal pain**	-	Mild – 1	-
**Constipation**	Mild – 1	-	-
**Diarrhea**	-	-	-
**Flatulence**	Mild – 1	Mild – 2	Mild – 1
**Abdominal bloating**	Mild – 2	Mild – 1	Mild – 1
			Moderate – 1
**Cramping**	-	-	-
**Heartburn**	-	-	Mild – 1
**After taste**	Mild – 6	Mild – 3	-
	Moderate – 1		

## Discussion

The current findings show, for the first time, that consumption of 3 g/d krill oil for 4 wk increases the plasma and RBC concentrations of total n-3 PUFA, EPA, and the sum of EPA and DHA compared with fish and corn oil in healthy humans. Krill oil consumption also resulted in a significant decrease in the total n-6:n-3 PUFA ratio and increased the omega-3 index compared with fish oil and control treatments. Fish oil treatment also significantly increased the plasma and RBC concentrations of total n-3 PUFA and EPA and decreased the total n-6:n-3 PUFA ratio compared with control. These results indicate that bioavailability of krill oil n-3 PUFA might be more pronounced compared with that of fish oil, likely due to the structural differences between these two marine oils. Moreover, both krill and fish oil were well tolerated by participants and caused no adverse effects which shows the safe nature of krill oil. According to our knowledge, the current study is the first to compare the effects of krill and fish oils on fatty acid profile in plasma and RBC after 4 wk of consumption, using a randomized crossover design.

In the present study, krill oil supplementation for 4 wk resulted in a substantial increase in EPA total n-3 PUFA and the sum of EPA and DHA levels in the plasma, compared with fish oil treatment although fish oil also increased the plasma EPA, DHA and total n-3 PUFA compared with control. Since both krill and fish oil treatments delivered the same amount of total n-3 PUFA, these findings indicate that the bioavailability of n-3 PUFA from krill oil might be higher than that of n-3 PUFA from fish oil. Several studies were carried out attempting to test the hypothesis that n-3 fatty acids will be better absorbed as PL based oil, such as krill oil, compared with TG based oil, such as fish oil, [10,17] or ethyl-esters [18]. However all of these trials, although using different study designs, duration, selection criteria and concentrations of the n-3 PUFA, consistently show a non-significant increased bioavailability of n-3 PUFA from krill oil compared with fish oil. It is very likely that the reason for the poor results presented thus far is due to use of study designs not suitable for absorption studies.

Krill oil supplementation reduced plasma n-6 PUFA, along with increases in n-3 PUFA concentrations, resulting in decreased n-6:n-3 PUFA ratio compared with fish oil treatment. Furthermore, fish oil intake also reduced the plasma n-6 PUFA and n-6:n-3 PUFA ratio compared with control. In the current study, the lower plasma n-6 PUFA and n-6:n-3 PUFA ratio with krill oil intake compared with fish oil intake might also be due to differences in fatty acid composition of the krill and fish oils used. The n-6 PUFA content of the krill oil was low (1.73 g/100 g oil) compared with fish oil (21.95 g/100 g oil). The current study did not control the n-6 PUFA concentrations between krill and fish oil. However, even with higher n-6 PUFA content, fish oil treatment actually significantly reduced the plasma concentrations of n-6 PUFA. Furthermore, n-6 PUFA consumption by the participants was not controlled although n-3 PUFA consumption was well controlled during the intervention period. Amounts of n-6 PUFA in the diet might be higher than the difference in the amount between the different treatment oils. Hence, it is understandable that consumption of both krill and fish oils resulted in reductions of n-6 PUFA which might also contribute in decreased n-6:n-3 ratio along with elevations of total n-3 PUFA concentration.

Reduction in n-6:n-3 PUFA ratio after krill oil consumption observed in the current study was in line with previous reports analyzing the n-6:n-3 ratio with intake of n-3 rich oils [10,17,19]. It has been shown that n-6:n-3 PUFA ratio is strongly associated with CVD risk [20]. Hence, krill oil supplementation could be recommended to increase the n-3 PUFA, omega-3 index and reduce the n-6:n-3 PUFA ratio thereby reducing the risk of CVD. Indeed, it is currently recommended that consumption of 1-3 g/d of EPA and DHA through fish oil reduces CVD risk [21,22]. However, evidence from epidemiological studies on effects of fish oil on CVD is inconsistent with totally negative association [3,23] or no effect [24,25] having been identified. Bioavailability of n-3 PUFA could play a major role for these discrepancies. It has been shown that DHA is highly absorbed when delivered by PL compared with fish oil in mice deprived of essential fatty acids [26]. Additional in vivo studies using animal models demonstrated that the level of absorption of DHA into target tissues, such as brain and heart, is affected by the source of the DHA. It was shown that PL-bound DHA deliver the DHA to brains hearts and liver in different animal models including mice, rats and baboons more efficiently than TG-bound DHA [14,26-28]. In humans, Carnielli et al. tested the ability of preterm babies to absorb DHA given in formulas enriched with DHA in PL form or TG forms [29]. The results showed that DHA in a PL form was absorbed significantly better compared than in a TG form.

Sum of EPA and DHA in RBC has been considered as the highly valuable omega-3 index and was demonstrated to serve as a risk factor for death from CVD [2,30,31]. In the current study, krill oil consumption resulted in an elevated omega-3 index compared with consumption of fish oil and control oil treatments, emphasizing the efficiency and higher bioavailability of krill oil over fish oil. Concentrations of EPA and DHA in RBC have a longer half-life of about 4–6 times compared with plasma EPA and DHA [32], thus reflecting long term intakes of n-3 PUFA status of individuals [2]. Measurement of fatty acids in RBC reflects the tissue composition and the concentrations of the EPA and DHA in RBC which are not influenced by dyslipidemia [30]. Increased n-3 PUFA concentrations in RBC have been shown to be associated with reduced risk of CVD [2,33-35].

In the current study, both krill and fish oil treatments failed to affect serum HDL and TG concentrations compared with control. No significant differences were seen with effects of krill oil compared with fish oil consumption on serum lipid levels. Indeed, both krill and fish oils increased serum total and LDL cholesterol concentrations compared with control. Results are not surprising as the participants used in the study were normolipidemic healthy individuals. Studies have shown that higher dosages of about 2–4 g/d of EPA and DHA were needed to bring significant reductions in serum lipid concentrations of normolipidemic participants [5,36,37]. Although krill oil supplementation has demonstrated hypolipidemic activity in some studies, this effect is found to be more pronounced with participants with hyperlipidemia [11,36,38]. Similar results were found in previous studies with normolipidemic participants in whom there was no effect on serum lipids [10,17,39]. Reduction of serum TG by n-3 PUFA also depends on their baseline concentrations [36,38,40]. Supplementation of 1–4 g/d of EPA reduced serum TG in hyperlipidemic participants compared with normolipidemics [41-44]. Consumption of n-3 PUFA from fish oil increased the diameter of LDL particles [45] and reduced the number of small dense LDL particles while increasing LDL particle size which could be the reasons for the increase in LDL in some studies [46,47].

In regards to safety parameters, krill oil supplementation of 4 wk was well tolerated, with participants experiencing no adverse events. No changes were seen in vital signs, anthropometric measures and hematological variables or concerns by the study physician after the physical examination between the different treatments. Also, treatment with krill oil did not change any of the gastrointestinal symptoms compared with fish oil or control intervention except mild burping and aftertaste. The findings on safety parameters along with the high compliance from participants indicate the safety of krill oil consumption as shown by other studies [10,17].

### Strengths of the study

Selection of a randomized, placebo controlled, double blinded crossover study design added robustness and increased the statistical power of the present trial. Dosages of n-3 PUFA for krill and fish oil treatments were kept identical which enables the direct comparison between the effects of krill and fish oil. Measurement of fatty acids in RBC reflects the tissue composition and is not influenced by dyslipidemic conditions. During the study, all participants kept their dietary habits and physical activities constant. None of the participants consumed any concomitant medications or supplementation or exceeded more than one fish or sea food serving per month throughout the study period which confirms that the changes in the circulatory fatty acid profile of the participants were only from the intervention, without any other dietary influence. Participants’ reported compliance with each treatment was above 96% which adds strength to the results and indicates the tolerability and acceptability of the treatment capsules by the participants. The physical examination of participants at baseline and endpoint by the study physician for safety and well-being and the weekly telephone monitoring for compliance and adverse events added strength to the study.

### Limitations of the study

The n-6 fatty acid concentration in fish oil was higher than krill oil which could influence the effects of n-3 fatty acids and the ratio between n-6 and n-3 fatty acids. Although we found a significant beneficial effect in fatty acid profile after krill oil consumption, the intervention period of each treatment phase was only 4 weeks long. In order to measure and achieve steady-state conditions with circulatory n-3 PUFA concentration, longer intervention periods could be recommended. Additionally, testing and comparing effects of krill oil and fish oil consumption over different time points would be very informative. The current study was a free living design which did not strictly control the macronutrient profile of each participant diet. In addition, n-3 PUFA consumption from sources other than marine foods was not strictly controlled although participants were recommended to keep their diets constant during the study. A full feeding control study with all background diets and n-3 PUFA well controlled could also be recommended.

In summary, the consumption of krill oil significantly increased plasma and RBC concentrations of EPA along with total n-3 PUFA levels; and decreased the n-6:n-3 PUFA ratio compared with fish oil and the control treatments. The results indicate that there might be a higher bioavailability of n-3 PUFA from krill oil over fish oil. Krill oil also efficiently increased the omega-3 index compared with fish oil and control. However, krill oil, similar to fish oil, consumption resulted in a slight but significant increase in total and LDL cholesterol without altering the HDL and TG, in a manner that no alterations were seen in the TC/HDL ratio. Overall, four wk consumption of krill oil was well tolerated, and could be more effective in increasing n-3 PUFA and omega-3 index than fish oil, with no indication of adverse effects.

### Participants and methods

#### ***Participant selection***

Healthy males and premenopausal non pregnant or nursing females (n = 24) ages 18–49 y were recruited by advertisement at the Richardson Centre for Functional Foods and Nutraceuticals on the University of Manitoba campus. Participants were screened and excluded if they reported any history of cancer, rheumatoid arthritis, chronic illness, cardiovascular problems, hepatobiliary and renal disease, diabetes mellitus, inflammatory bowel disease, pancreatitis, neurological/psychological disease, bleeding disorders, experienced platelet abnormalities, and gastrointestinal disorders that could interfere with fat absorption. In addition, participants were excluded if they admitted an allergy to fish or sea foods, or reported consuming supplements including n-3 PUFA in the past 6 mo or consuming more than one fish serving per mo during the mo prior to the start of the study. Participants were included if they were nonsmokers, with serum TG levels less than 200 mg/dL, total cholesterol less than 240 mg/dL, LDL-cholesterol less than 160 mg/ dL, BMI less than 28, not consuming more than one alcoholic drink/d and not taking any medications that would interfere with lipid metabolism or to control blood lipids or treat hypertension. The protocol of the study with all procedures was reviewed and approved by the Human Ethical Review Committee of the University of Manitoba (B2011: 014). All participants were explained the study protocol and written consent was obtained.

#### ***Study design and intervention***

**Figure 1:**
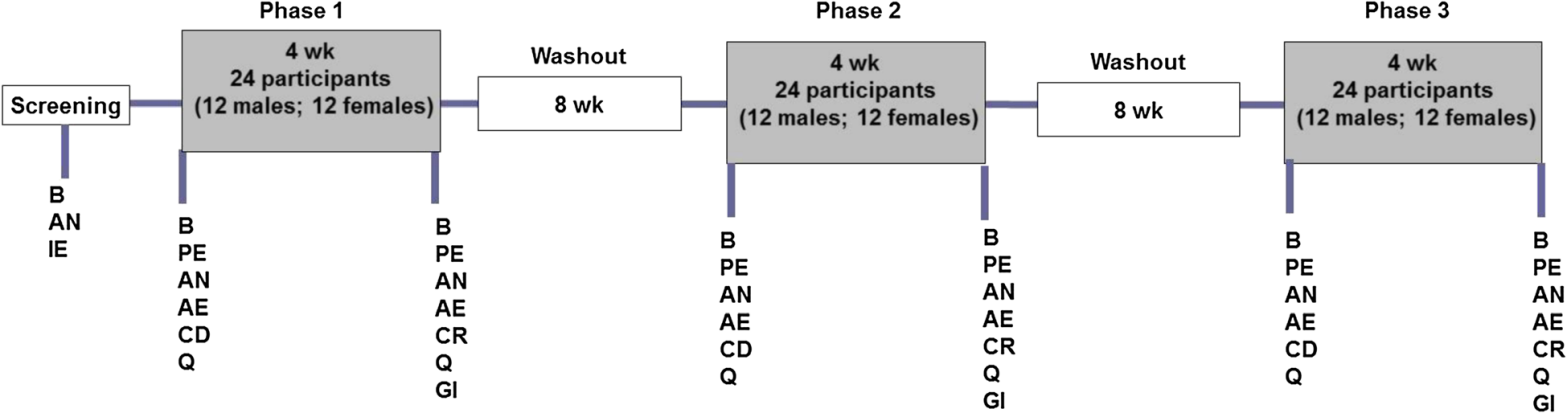
**Schematic representation of the experimental protocol.** B - 12 h fasted blood collection; AN - Anthropometric measurements; IE - Inclusion and Exclusion criteria; PE - Physician examination; AE - Adverse events; CD - Capsule dispensation (KO or FO or corn oil capsules); CR - Left over capsules return; Q - Questionnaires (Fish and sea food consumption, concomitant medications); GI - Gastro intestinal questionnaires.

The study design was a randomized, placebo controlled, double blinded, crossover, conducted to comply with Good Clinical Practice guidelines and in accordance with the Helsinki declaration of 1975 as revised in 1983. The study included three treatment periods. Each treatment phase lasted 4 wk and was separated by washout phases of 8 wk (Figure 1). Washout period of 8 wk was chosen based on results of a previous human trial with n-3 fatty acid supplementation [48]. Stratified randomization was used to allocate participants to the 3 possible treatment sequences so that an equal number of men and women were allocated to each sequence. Interventions were conducted during May and December 2011. Participants were provided with identical looking capsules containing krill, fish or corn oil on the first d of each treatment phase. During each treatment period, participants consumed 6 capsules per d, 3 in the morning and 3 at night along with their meals and each capsule consisting of 500 mg of one of the oils (a total of 3 g which were taken in six 500 mg capsules, krill oil (K•REAL™) was supplied by Enzymotec Ltd. and fish oil was Omevital TG 18/12 supplied by Napro Pharma, Norway). Fatty acid composition of krill, fish and corn oils are shown in Table 6. Daily doses of both krill and fish oil treatments provided 600 mg of n-3 PUFA. However, only krill oil capsules delivered 1800 μg/d of astaxanthin in addition to the fatty acids.

**Table 6:** Fatty acid composition of krill, fish and corn oil used in the study

**Fatty acid (% of total fatty acids)**	**Krill oil**	**Fish oil**	**Corn oil**
C12 (Lauric)	0.27	0.16	0.13
C14 (Myristic)	11.82	4.49	0.00
C14:1 (Myristoleic)	0.16	0.12	0.00
C15 (Pentadecanoatenoic)	0.47	0.35	0.00
C16 (Palmitic)	22.11	17.06	15.01
C16:1 (Palmitoleic)	6.09	6.53	0.00
C17 (Margaric)	2.02	0.38	0.00
C18 (Stearic)	1.39	3.54	3.31
C18:1n9 (Oleic)	13.28	2.62	1.08
C18:1n7 (Vaccenic)	7.56	0.00	0.00
C18:2n6 (Linoleic)	2.06	32.49	77.57
C18:3n6 (gamma-Linolenic)	0.00	0.24	0.00
C18:3n3 (alpha-Linolenic)	0.93	1.28	1.61
C18:4n3 (Moroctic)	2.70	2.67	0.00
C20 (Arachidic)	0.00	0.50	0.57
C20:1n9 (Eicosenoic)	0.80	1.04	0.44
C20:2n6 (Eicosadienoic)	0.41	0.00	0.00
C20:4n6 (Arachidonic)	0.40	0.65	0.00
C20:4n3 (Eicosatetraenoic)	0.26	0.80	0.00
C20:5n3 (Eicosapentaenoic)	16.44	13.46	0.00
C22 (Behenic)	0.00	0.73	0.27
C22:1n9 (Euricic)	0.56	0.00	0.00
C22:2n9 (Docosadienoic)	0.43	0.50	0.00
C22:5n3 (Docosapentaenoic)	0.38	1.36	0.00
C22:6n3 (Docosahexaenoic)	9.48	8.66	0.00
C24:1n9 (Nervonic)	0.00	0.35	0.00
Total n-3 PUFA	30.18	28.24	1.61
Total n-6 PUFA	2.86	33.38	77.57
Total n-9 PUFA	15.07	4.51	1.52
Total PUFA	33.47	62.11	79.18
Total MUFA	28.46	10.67	1.52
Total SFA	38.07	27.22	19.30

Body weight, blood pressure, waist and hip circumference measurements and physical examinations were performed at baseline and endpoint of each treatment following standard procedures [49]. Gastrointestinal tolerability and well-being questionnaires were assessed at the endpoint of each treatment. Blood samples after 12 h fasting were collected at baseline and endpoint of each phase. Samples were centrifuged at 3000 rpm for 20 min followed by separation of plasma and RBC and aliquots were stored in -80 degrees C until analysis. A weekly telephone questionnaire was completed by study coordinators to monitor treatment adherence and safety during the study period. Questions included; capsule intake, fish or sea food intake, adverse events and concomitant medication use. In addition, participants recorded their capsule consumption and remaining capsules were returned at the end of each phase. Compliance was calculated using the number of remaining capsules returned and written records kept by the participants.

#### ***Plasma and RBC fatty acid analyses***

Plasma and RBC total lipids were extracted using the Folch method [50] which involved chloroform-methanol (2:1, v/v) containing 0 · 01% BHT (Sigma-Aldrich, Oakville, ON, Canada) and heptadecanoic acid as an internal standard (Sigma-Aldrich, Oakville, ON, Canada). Extracted fatty acids were methylated with methanolic HCl. Fatty acid methyl esters were separated on a Supelcowax 10 column (30 m X 0 · 25 mm with 0 · 25 mm film thickness; Supelco, Bellefonte, PA, USA) using a gas chromatograph equipped with a flame ionisation detector (Bruker 430). The oven was programmed from 70 to 240 degrees C with the following temperature steps (70 degrees C for 2 min, rise of 30 degrees C/min, 180 degrees C for 1 min, rise of 10 degrees/min, 200 degrees C for 2 min, rise of 2 degrees C/min, 220 degrees C for 4 min, rise of 20 degrees/min, 240 degrees for 6 min). Samples were analyzed with a 20:1 split ratio; helium was used as the carrier gas with a column flow rate of 1.0 ml/min. Temperatures of the injector and detector were set at 270 and 290 degrees C, respectively. Individual fatty acids were identified by comparison with known standards (NuChek Prep, Inc., Elysian, MN, USA). Individual fatty acids were calculated according to the peak area relative to the total area and expressed as the percentage of total fatty acids.

#### ***Serum lipid profile and blood cells count analyses***

Serum lipid profile including total and HDL-cholesterol and TG levels were measured using a Vitros 350 Autoanalyser (Orthoclinical diagnostics). LDL-cholesterol levels were calculated using Friedewald equation [51]. Complete blood counts were also determined using a Beckmann coulter LH780 at baseline and at endpoint of each treatment phase.

#### ***Statistical analysis***

Differences between krill oil, fish oil, and placebo control interventions were analyzed for each dependent measure. Friedman Test was used to test for significant differences between treatment groups regarding the results in baseline, end-point and change between baseline and end-point. Data were analyzed using SAS statistical software, version 9.2. Values were expressed as mean ± STD and p values <0.05 were considered significant. Safety data were examined descriptively for each arm and compared across treatments. Furthermore, statistical analyses such as Kruskal-Wallis and Friedman tests were used to identify significant differences between different phases regarding baseline result for each treatment separately and if significant differences existed between phases regarding the baseline measures (ignoring the treatment effect). These tests were done to determine the efficiency of the washout period.

## Abbreviations

AHA: American heart association; CVD: Cardiovascular disease; DHA: Docosahexaenoic acid; EPA: Eicosapentaenoic acid; FDA: Food and drug administration; GI: Gastrointestinal; GRAS: Generally regarded as safe; HDL: High-density lipoproteins; LDL: Low-density lipoproteins; MCHC: Mean corpuscular hemoglobin concentration; MCH: Mean corpuscular hemoglobin; MCV: Mean corpuscular volume.

## Competing interests

VRR and PJHJ declare no conflict of interest; IE and SZ are employees of Enzymotec Ltd.

## Authors contributions

The Authors’ responsibilities are as follows: IE and SZ designed the study and wrote the protocol; VRR and PJHJ coordinated and completed the trial and collected all the data, analyzed the data and statistics; VR, PJHJ, IE, SZ wrote the final draft and had primary responsibility for the final conduct. All authors read and approved the final manuscript.

## Supplementary Material

Additional file 1: Table S1 Plasma secondary fatty acids composition in individuals after interventions. **Table S2**: RBC secondary fatty acids composition in individuals after interventions. Click here for file
