# Supplementary material for: Enhanced increase of omega-3 index in healthy individuals with response to 4-week n-3 fatty acid supplementation from krill oil versus fish oil
Source: Lipids Health Dis. 2013 Dec 5;12:178. doi: 10.1186/1476-511X-12-178 (PMC4235028; doi:10.1186/1476-511X-12-178)
Supplement: Additional file 1: Table S1 — Plasma secondary fatty acids composition in individuals after interventions. Table S2: RBC secondary fatty acids composition in individuals after interventions. [file 1476-511X-12-178-S1.doc]

**Additional file 1: Table S1. Plasma secondary fatty acids composition in individuals after interventions**

| **Fatty Acid** | **Treatment** | **Mean** | **STD** | **Median** | **p-values^1^** |
| --- | --- | --- | --- | --- | --- |
| **C14:0** | Krill oil | 0.62 | 0.17 | 0.61 | 0.1448 |
|  | Fish oil | 0.55 | 0.21 | 0.54 |  |
|  | Corn oil | 0.56 | 0.16 | 0.56 |  |
| **C16:0** | Krill oil | 29.25 | 1.62 | 29.00 | 0.8964 |
|  | Fish oil | 29.62 | 1.67 | 29.83 |  |
|  | Corn oil | 28.80 | 1.91 | 28.43 |  |
| **C16:1n7** | Krill oil | 1.04 | 0.34 | 0.99 | 0.9592 |
|  | Fish oil | 1.04 | 0.27 | 1.07 |  |
|  | Corn oil | 1.04 | 0.25 | 1.01 |  |
| **C18:0 (SA)** | Krill oil | 11.30 | 1.23 | 11.57 | 0.5775 |
|  | Fish oil | 11.34 | 1.11 | 11.19 |  |
|  | Corn oil | 11.08 | 1.06 | 11.31 |  |
| **C18:1n9 (OA)** | Krill oil | 13.57 | 2.39 | 12.91 | 0.1448 |
|  | Fish oil | 13.21 | 1.71 | 13.04 |  |
|  | Corn oil | 14.26 | 2.22 | 14.61 |  |
| **C18:2n6 (LA)** | Krill oil | 22.17* | 2.99 | 23.11 | 0.0054 |
|  | Fish oil | 22.11* | 2.47 | 22.39 |  |
|  | Corn oil | 23.72 | 2.54 | 23.96 |  |
| **C18:3n6** | Krill oil | 0.11 | 0.05 | 0.11 | 0.8964 |
|  | Fish oil | 0.11 | 0.09 | 0.09 |  |
|  | Corn oil | 0.13 | 0.11 | 0.10 |  |
| **C18:3n3 (ALA)** | Krill oil | 0.57 | 0.15 | 0.55 | 0.9592 |
|  | Fish oil | 0.58 | 0.22 | 0.55 |  |
|  | Corn oil | 0.60 | 0.16 | 0.57 |  |
| **C:20:0** | Krill oil | 0.38 | 0.04 | 0.37 | 0.1448 |
|  | Fish oil | 0.41 | 0.06 | 0.41 |  |
|  | Corn oil | 0.38 | 0.05 | 0.38 |  |
| **C20:1n9** | Krill oil | 0.32 | 0.19 | 0.26 | 0.8398 |
|  | Fish oil | 0.30 | 0.17 | 0.26 |  |
|  | Corn oil | 0.31 | 0.24 | 0.26 |  |
| **C20:2n6** | Krill oil | 0.27 | 0.07 | 0.26 | 0.9523 |
|  | Fish oil | 0.25 | 0.06 | 0.24 |  |
|  | Corn oil | 0.26 | 0.08 | 0.26 |  |
| **C20:3n6** | Krill oil | 1.96 | 0.61 | 1.87 | 0.8398 |
|  | Fish oil | 1.94 | 0.72 | 1.82 |  |
|  | Corn oil | 2.02 | 0.61 | 1.88 |  |
| **C20:4n6 (AA)** | Krill oil | 8.73 | 1.52 | 8.61 | 0.2063 |
|  | Fish oil | 9.15 | 1.51 | 8.87 |  |
|  | Corn oil | 9.34 | 1.94 | 8.89 |  |
| **C22:0** | Krill oil | 0.79 | 0.16 | 0.76 | 0.1448 |
|  | Fish oil | 0.90 | 0.15 | 0.89 |  |
|  | Corn oil | 0.83 | 0.16 | 0.83 |  |
| **C22:4n6** | Krill oil | 0.23* | 0.27 | 0.18 | 0.0018 |
|  | Fish oil | 0.21* | 0.06 | 0.19 |  |
|  | Corn oil | 0.25 | 0.06 | 0.24 |  |
| **C24:0** | Krill oil | 0.69 | 0.48 | 0.57 | 0.1448 |
|  | Fish oil | 0.84 | 0.54 | 0.69 |  |
|  | Corn oil | 0.88 | 1.20 | 0.63 |  |
| **C24:1n9** | Krill oil | 1.37 | 0.26 | 1.38 | 0.1448 |
|  | Fish oil | 1.51 | 0.25 | 1.49 |  |
|  | Corn oil | 1.36 | 0.39 | 1.23 |  |

Values are expressed as percentage of total fatty acids (n=24). ^1^ Friedman Test was used to test significant difference between treatment groups regarding the results in end-point. ^*^ indicates significant difference compared with corn oil treatment

**Additional file 1: Table S2. RBC secondary fatty acids composition in individuals after interventions**

| **Fatty Acid** | **Treatment** | **Mean** | **STD** | **Median** | **p-values^1^** |
| --- | --- | --- | --- | --- | --- |
| **C14:0** | Krill oil | 0.69 | 0.26 | 0.62 | 0.2336 |
|  | Fish oil | 0.68 | 0.27 | 0.60 |  |
|  | Corn oil | 0.54 | 0.29 | 0.43 |  |
| **C16:0** | Krill oil | 25.67 | 1.33 | 25.47 | 0.1721 |
|  | Fish oil | 26.47 | 1.37 | 26.30 |  |
|  | Corn oil | 25.74 | 1.00 | 25.55 |  |
| **C16:1n7** | Krill oil | 0.63 | 0.27 | 0.61 | 0.9592 |
|  | Fish oil | 0.65 | 0.23 | 0.64 |  |
|  | Corn oil | 0.65 | 0.26 | 0.59 |  |
| **C18:0 (SA)** | Krill oil | 12.48 | 1.43 | 12.43 | 0.6274 |
|  | Fish oil | 12.75 | 1.40 | 12.70 |  |
|  | Corn oil | 12.66 | 1.31 | 12.67 |  |
| **C18:1n9 (OA)** | Krill oil | 13.27 | 1.06 | 13.0 | 0.6601 |
|  | Fish oil | 13.20 | 0.95 | 13.08 |  |
|  | Corn oil | 13.38 | 0.86 | 13.29 |  |
| **C18:2n6 (LA)** | Krill oil | 11.39 | 1.20 | 11.31 | 0.2336 |
|  | Fish oil | 11.95 | 1.49 | 11.60 |  |
|  | Corn oil | 11.98 | 1.15 | 11.79 |  |
| **C18:3n6** | Krill oil | 0.15 | 0.12 | 0.11 | 0.1721 |
|  | Fish oil | 0.09 | 0.05 | 0.08 |  |
|  | Corn oil | 0.10 | 0.07 | 0.10 |  |
| **C18:3n3 (ALA)** | Krill oil | 0.35 | 0.11 | 0.34 | 0.5294 |
|  | Fish oil | 0.33 | 0.14 | 0.28 |  |
|  | Corn oil | 0.36 | 0.12 | 0.35 |  |
| **C:20:0** | Krill oil | 0.42 | 0.09 | 0.41 | 0.8963 |
|  | Fish oil | 0.41 | 0.07 | 0.39 |  |
|  | Corn oil | 0.41 | 0.07 | 0.41 |  |
| **C20:1n9** | Krill oil | 1.19 | 2.08 | 0.47 | 0.8963 |
|  | Fish oil | 0.69 | 0.52 | 0.49 |  |
|  | Corn oil | 0.75 | 0.58 | 0.56 |  |
| **C20:2n6** | Krill oil | 0.25 | 0.03 | 0.25 | 0.1721 |
|  | Fish oil | 0.26 | 0.04 | 0.25 |  |
|  | Corn oil | 0.27 | 0.04 | 0.27 |  |
| **C20:3n6** | Krill oil | 1.37 | 0.37 | 1.28 | 0.2336 |
|  | Fish oil | 1.40 | 0.36 | 1.26 |  |
|  | Corn oil | 1.44 | 0.35 | 1.40 |  |
| **C20:4n6 (AA)** | Krill oil | 13.49 | 1.64 | 13.42 | 0.1233 |
|  | Fish oil | 13.32 | 1.57 | 13.72 |  |
|  | Corn oil | 14.04 | 1.66 | 14.03 |  |
| **C22:0** | Krill oil | 1.11 | 0.33 | 1.14 | 0.4497 |
|  | Fish oil | 1.16 | 0.24 | 1.16 |  |
|  | Corn oil | 1.19 | 0.24 | 1.13 |  |
| **C22:4n6** | Krill oil | 2.94 | 0.51 | 2.90 | 0.2336 |
|  | Fish oil | 2.91 | 0.60 | 2.92 |  |
|  | Corn oil | 3.07 | 0.82 | 3.03 |  |
| **C24:0** | Krill oil | 2.75 | 0.53 | 2.72 | 0.8963 |
|  | Fish oil | 2.76 | 0.65 | 2.82 |  |
|  | Corn oil | 2.80 | 0.59 | 2.70 |  |
| **C24:1n9** | Krill oil | 3.46 | 0.64 | 3.33 | 0.2336 |
|  | Fish oil | 3.28 | 0.91 | 3.35 |  |
|  | Corn oil | 3.53 | 0.58 | 3.61 |  |

Values are expressed as percentage of total fatty acids (n=24). ^1^ Friedman Test was used to test significant difference between treatment groups regarding the results in end-point.
